# In Hepatocellular Carcinoma, miRNA-296-3p Targets MSL2 and Suppresses Cell Proliferation and Invasion

**DOI:** 10.1155/2021/7430468

**Published:** 2021-12-02

**Authors:** Xiaocui Li, Min An, Zhenjun Gao

**Affiliations:** Department of Gastroenterology, Qingpu Branch of Zhongshan Hospital Affiliated to Fudan University, 1158 Park Road(E), Qingpu, Shanghai 201700, China

## Abstract

Hepatocellular carcinoma (HCC) is the third-highest cause of cancer-related death in the world. miRNAs have a role in cell division, differentiation, and death biological processes. They are typically dysregulated in cancers, affecting tumor progression. miRNA-296-3p appears to play a crucial role in cancer control, according to new research. However, its expression and roles in HCC are unknown. This study used qRT-PCR and western blotting to detect the miRNA-296-3p and male-specific lethal 2 (MSL2) expression. In addition, cell proliferation, migration, invasion, and apoptosis were studied using CCK-8, flow cytometric analysis, colony formation assay, wound healing test, and transwell assays. The results show that miRNA-296-3p is underexpressed in HCC cell lines, particularly in Huh-7 and HepG2 cells. miRNA-296-3p overexpression lowers the ability of HCC cells to proliferate, migrate, and invade while increasing cell death. Luciferase reporter experiments revealed that the MSL2 is a direct target of miRNA-296-3p. Furthermore, overexpression of miRNA-296-3p reduced MSL2 mRNA and protein levels considerably, according to our findings. Furthermore, the rescue experiments showed that the MSL2 overexpression partially blocked the inhibition effects of miRNA-296-3p mimic on the proliferation and migration of HCC cells. The above results show that miRNA-296-3p may have a repressive effect in HCC by targeting MSL2 and could be used as a therapeutic target for HCC treatment.

## 1. Introduction

Hepatocellular carcinoma (HCC) is the second leading cause of cancer-associated death worldwide [1–3]. Despite the significant advancements achieved in HCC treatment, the current survival rate of HCC patients remains poor [4, 5]. Consequently, to improve HCC treatment and the quality of patients' lives, it is critical to investigate the molecular mechanisms behind HCC progression and look for new HCC treatment targets.

MicroRNAs (miRNAs), short noncoding RNAs of 18–25 nucleotides, can attach to the 3′-untranslated regions (3′-UTRs) of target mRNAs to cause mRNA degradation and translation inhibition [6–8]. It is reported that miRNAs play an essential role in tumor occurrence and development [9–11], especially in the progression of HCC [12–14]. miRNA-296 has two transcripts, miRNA-296-3p and miRNA-296-5p, which have been involved in tumor development [15, 16]. Ge et al. found that miR-296-3p could play a tumor suppressor role in NSCLC both in vitro and in vivo and first reported that miR-296-3p could regulate the migration and invasion of A549 cells by targeting RABL3 [17]. Furthermore, in glioblastoma, miR-296-3p suppresses cell growth and promotes chemosensitivity [18]. miR-296-5p is also reported to be downregulated in HCC and inhibits EMT-related metastasis [19]. However, the significance of miRNA-296-3p in HCC and the underlying molecular mechanism remain unknown.

According to the previous research, chronic hepatitis B virus (HBV) infection is a significant risk factor for HCC [20–22]. Gao et al. [23] reported that MSL2 is extensively expressed in clinical HBV-related HCC tissues, implying that it could be a key mediator in the HBV-host interaction. Given the inhibition effects of miRNA-296-3p on tumors and the interaction of MSL2 and HBV, we assumed that miRNA-296-3p and MSL2 were involved in the procession of HCC. However, the interaction between miRNA-296-3p and MSL2 was not yet well documented.

Our study first identified the miRNA-296-3p expression characteristics in HCC. It confirmed the miRNA-296-3p beneficial effects on the proliferation, apoptosis, migration, and invasion of HCC cells. Second, we discovered that miRNA-296-3p had MSL2 as a target gene. Finally, we demonstrated that overexpression of MSL2 rescued the inhibition effect of miRNA-296-3p overexpression on HCC progression. In summary, our study revealed that miRNA-296-3p inhibited cell proliferation and invasion by targeting MSL2 in HCC cells and provided novel therapeutic targets against HCC.

## 2. Materials and Methods

### 2.1. Cell Culture and Transfection

The Chinese Academy of Sciences (CAS; Shanghai, China) provided the average human liver cells HL-7702. The American Type Culture Collection provided the human HCC cell lines Huh-7, MHCC97-H, HepG2, and SMMC-7721 (ATCC; Manassas, VA, USA). All cell lines were grown in Dulbecco's modified Eagle's medium (DMEM) containing 10% fetal bovine serum (FBS) and 1% antibiotics (penicillin 100 IU/mL and streptomycin) (Gibco, Grand Island, NY, USA). These cells were incubated at 37°C in a humidified environment containing 5% CO_2_.

Negative control (NC mimic), miR296-3p mimic, si-NC, and si-MSL2 were purchased from GenScript. First, seeded cells were grown to 70–80% confluence in 6-well, 24-well, or 96-well plates. Then, plasmids (1 *μ*g) and negative control (NC mimic), miR296-3p mimic, si-NC, and si-MSL2 were transfected into cells. After 48 h, the cells were harvested for further *in vitro* investigations. All the Huh-7 and HepG2 cell transfections used Lipofectamine ® 2000 (Invitrogen, CA, USA).

### 2.2. qRT-PCR Assay

Total RNA was extracted from the different cells using TRIzol reagent (Invitrogen, CA, USA), and RNA was reverse-transcribed to cDNA. The expression of miRNA was detected by the Hairpin-it™ MicroRNAs Quantitation Kit (GenePharma, Shanghai, China) and the SYBR green reagents (Vazyme, Nanjing, China). The thermocycling conditions were as follows: 10 min at 95°C for 1 cycle, followed by denaturation at 95°C for 30 sec, annealing at 56°C for 1 min, and final extension at 72°C for 30 sec for 40 cycles. The relative expression levels of miRNAs and mRNAs were evaluated using the 2^−ΔΔCq^ method [24], with U6 or GAPDH as the internal references. The following were the sequences of specific primers: miRNA-296-3p-F: 5′-TGGGAGGGCCCCCCCTCAA-3′, R: 5′-TGGTGTCGTGGAGTCG-3′; U6-F:5′-GTGATCACTCCCTGCCTGAG-3′, R: 5′-GGACTTCACTGGACCAGACG-3′; MSL2-F: 5′-ACAGTGAGAAAGTTCAGCCA-3′, R: 5′-AGCACGCCCACATTTACA-3′; and GAPDH-F: 5′-TGCCAAATATGATGACATCAAGAA-3′, R: 5′-GGAGTGGGTGTCGCTGTTG-3′.

### 2.3. Western Blotting Assay

Total proteins were extracted from cells in RIPA buffer with protease inhibitor and then quantified using a BCA protein assay kit (Pierce, Santa Cruz, CA, USA). SDS-PAGE was used to separate equivalent amounts of protein and then deposited onto PVDF membranes (Millipore, Billerica, USA). After blocking with 5% nonfat milk, the primary antibodies were incubated with the PVDF membrane overnight at 4°C, followed by 1 h at 25°C with the appropriate secondary antibody. Enhanced chemiluminescence was used to see protein bands (Millipore, Billerica, MA, USA). Proteintech provided the primary antibodies against Cyclin D1, CDK2, P21, and *β*-actin (Chicago, IL, USA). Abcam provided antibodies against PCNA, Cox-2, MMP-2, and MMP-9 (Cambridge, MA, USA). Novus Biologicals provided the antibody against MSL2 (Littleton, CO, USA). *β*-Actin was used as the internal reference. Protein bands were visualized using enhanced chemiluminescence (MilliporeSigma) and quantified using ImageJ software (version 4.3; National Institutes of Health).

### 2.4. Cell Counting Kit-8 (CCK-8) Assay

The CCK-8 kit was used to assess the ability of cells to multiply (Beyotime, Shanghai, China). Cells (3000 cells/well) were seeded in 96-well plates and transfected with miRNA-296-3p mimic or NC mimic for 24, 48, and 72 hours, after which they were incubated in the dark for 2 hours with CCK-8 solution (10 *μ*L). An ultraviolet spectrophotometer was used to measure optical density (OD) values at 450 nm (Thermo Fisher Scientific, USA).

### 2.5. Flow Cytometric Analysis of Apoptosis Rate and Cell Cycle

Flow cytometric analysis was used to evaluate cell apoptosis and the cell cycle. The transfected cells were simply rinsed twice with PBS before being collected with trypsin. The cells were then resuspended and centrifuged at 4°C for 5 minutes at 1000 rpm, and the supernatant was discarded. After that, the cells were fixed in 75% ethanol and stored at 20°C overnight. Finally, the cells were rinsed with PBS and incubated with RNase before being stained with propidium iodide (PI) reagent (Sigma-Aldrich, USA) for 15 minutes at room temperature in the dark. According to the manufacturer's recommendations, flow cytometry (Beckman Coulter, CA, USA) was used to detect the cell cycle or apoptosis.

### 2.6. Colony Formation Assay

The cells that had received different treatments were incubated in a six-well plate (500 cells/well) for the colony formation assay. The cells were rinsed twice in PBS, fixed with 10% formaldehyde, and stained for 10 minutes with 0.1% crystal violet. Then, the colonies (>50 cells) were counted and photographed (Olympus, ×40, Tokyo, Japan).

### 2.7. Wound Healing Assay

The ability of cells to migrate was assessed using a wound healing experiment. First, Huh-7 or HepG2 cells were sown on six-well plates, and a scratch was made using a 100 L pipette tip when the cells reached about 80% confluency. After that, the floating cells were rinsed multiple times with PBS, and a new medium was introduced. Next, we imaged the exact position of the wounds at two time points (0 and 48 h) with a microscope (Olympus, Tokyo, Japan). Finally, the migrated distance was calculated using a standard caliper.

### 2.8. Transwell Assay

The transwell chambers with 8 *μ*m pores (Merck KGaA, Darmstadt, Germany) were inserted into a 24-well plate to access the cells' migration and invasion. Cells were seeded onto the upper chamber with serum-free DMEM, while DMEM (10% FBS) was added to the lower chamber. For determining miRNA-296-3p overexpression effects on HCC cell migration or invasion, Huh-7 and HepG2 cells were transfected with miRNA-296-3p or NC mimic for 24 h. Nonmigrated cells on the upper surface of the membrane were removed with cotton swabs after a 24-hour incubation at 37°C, and migrated or invaded cells were fixed with 4% paraformaldehyde for 10 minutes before being stained with 0.1% crystal violet for 30 minutes. Finally, cells were counted in five random fields using an Olympus microscope (Tokyo, Japan).

### 2.9. Luciferase Reporter Assay

Complementary sequences between miRNA-296-3p and MSL2 were predicted using TargetScan 7.0 (https://www.targetscan.org/vert_72/). MSL2 wild-type or mutant type was cotransfected with miRNA-296-3p mimics or the control into HEK-293T cells using Lipofectamine 2000 (Invitrogen, CA, USA). The Dual-Luciferase Reporter Assay Kit was used to measure luciferase activity 48 hours after transfection (Beyotime, Shanghai, China).

### 2.10. Statistical Analysis

Every experiment was carried out at least three times. The mean and standard deviation were used to present all of the data (SD). The significance between the two groups was determined using the Student's *t*-test; statistical analysis was performed using one-way ANOVA. The comparison between the two groups was done with a post hoc test for data analysis among multiple groups. ^*∗*^*P* < 0.05,  ^*∗∗*^*P* < 0.01 were considered statistically significant differences.

## 3. Results

### 3.1. miRNA-296-3p Is a Low-Expression Gene in HCC That Inhibits Cell Proliferation When Overexpressed

**Figure 1:**
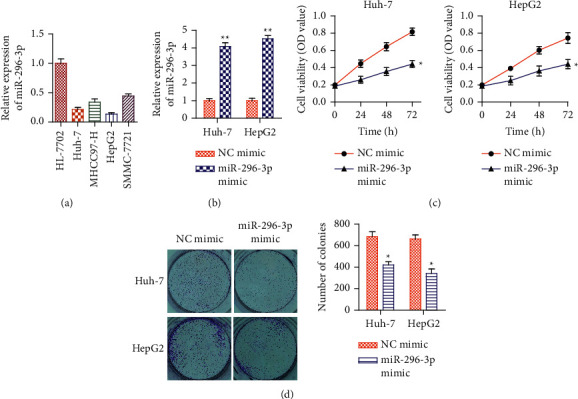
miRNA-296-3p levels and the effects of miRNA-296-3p mimic on HCC proliferation. (a) The expression level of miRNA-296-3p in normal human hepatocytes (HL-7702) and HCC cell lines (Huh-7, MHCC97-H, HepG2, and SMMC-7721) (qRT-PCR). ^*∗∗*^*P* < 0.01*vs*. HL-7702. *n* = 6. (b) The Huh-7 and HepG2 cells were transfected with miRNA-296-3p mimics and a negative control (NC mimic). The qRT-PCR assay was used to determine the miRNA-296-3p expression levels in each group. (c) CCK-8 kits evaluated cell proliferation and (d) cell growth and colony formation assays in Huh-7 and HepG2 transfected with NC mimic and miRNA-296-3p mimic. Values were mean ± SE, ^*∗*^*P* < 0.05,  ^*∗∗*^*P* < 0.01*vs*. NC mimic. *n* = 3 per group.

Firstly, to analyze the role of miRNA-296-3p in HCC, we detected normal human liver cells (HL-7702) and 4 HCC cell lines (Huh-7, MHCC97-H, HepG2, and SMMC-7721) and the expression level of miRNA-296-3p with qRT-PCR. The results revealed that HCC cell lines had much lower levels of miRNA-296-3p expression than HL-7702 cells (Figure 1(a)). As a result, Huh-7 and HepG2 cells were used in the following tests. Next, we transfected miRNA-296-3p mimic into Huh-7 and HepG2 cells to investigate the effects of miRNA-296-3p on HCC progression. The expression level of miRNA-296-3p in the miRNA-296-3p mimic was considerably upregulated, according to the qRT-PCR study after transfection (Figure 1(b)). Furthermore, the CCK-8 test and colony formation assay revealed that overexpression of miRNA-296-3p decreased proliferation in Huh-7 and HepG2 cells (Figures 1(c) and 1(d)), implying that miRNA-296-3p inhibits HCC cell proliferation.

### 3.2. Overexpression of miRNA-296-3p Promotes HCC Cell Cycle

**Figure 2:**
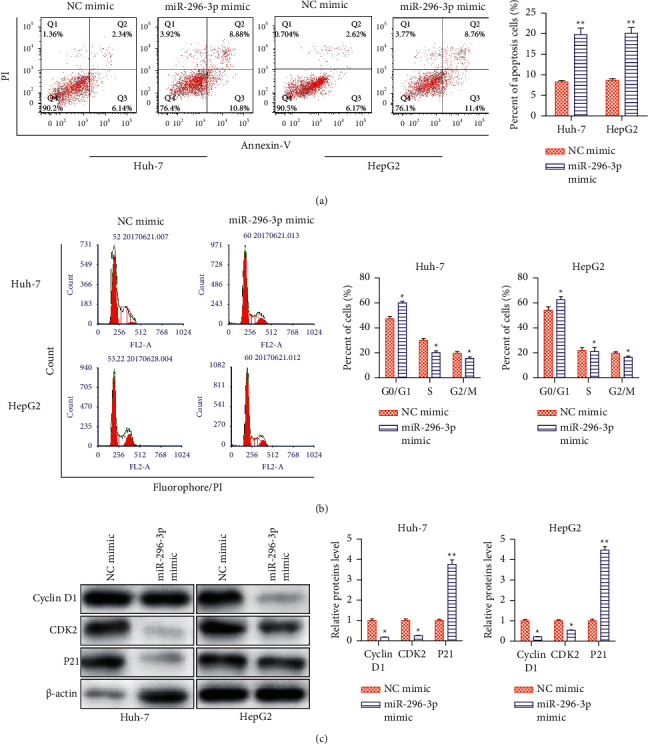
Effects of miRNA-296-3p overexpression on HCC apoptosis. Huh-7 and HepG2 cells were transfected with miRNA-296-3p mimic. (a) Flow cytometry analysis of the apoptosis rate. (b) Flow cytometry assays for the cell cycle. (c) Western blot analysis of the expression levels of cell cycle-associated proteins (cyclin D1, CDK2, and P21). Values were mean ± SE, ^*∗*^*P* < 0.05,  ^*∗∗*^*P* < 0.01*vs*. NC mimic. *n* = 3 per group.

miRNA-296-3p mimics dramatically accelerated apoptosis of Huh-7 and HepG2 cells, as demonstrated in Figure 2(a) compared to the NC-mimic group. Furthermore, flow cytometry analysis revealed that overexpression of miRNA-296-3p resulted in a considerable increase in G0/G1 phase cell numbers and a decrease in S or G2/M phase cell numbers (Figure 2(b)). Compared to the NC-mimic group, miRNA-296-3p mimics dramatically accelerated apoptosis of Huh-7 and HepG2 cells, as seen in Figure 2(a). According to flow cytometry studies, overexpression of miRNA-296-3p also resulted in a significant increase in G0/G1 phase cell numbers and a decrease in S or G2/M phase cell counts (Figure 2(b)).

### 3.3. miRNA-296-3p Negatively Regulates the Migration and Invasion of HCC Cells

**Figure 3:**
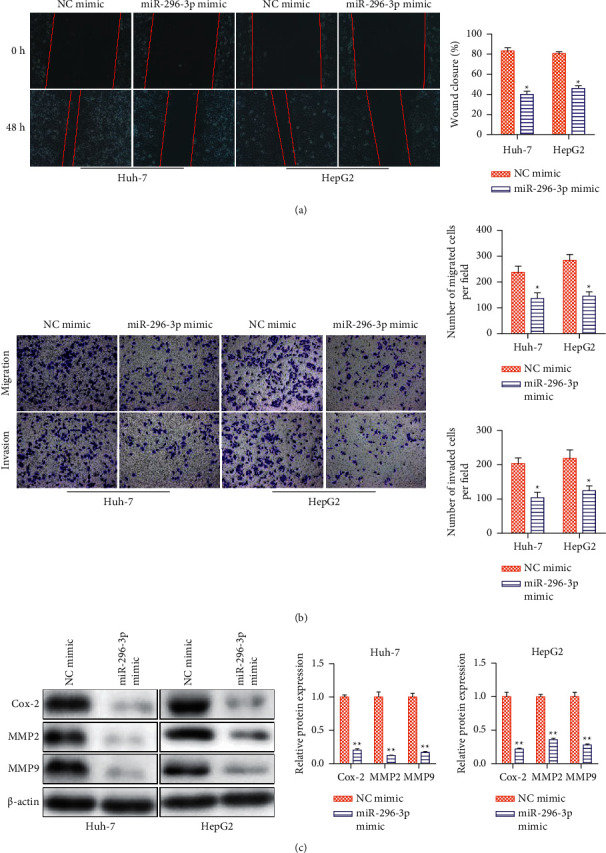
miRNA-296-3p negatively regulates the migration and invasion of HCC cells. Huh-7 and HepG2 cells were treated with NC mimic and miRNA-296-3p mimic. (a) Wound healing assay. Representative pictures are shown at 0 and 48 h after the wound was made. (b) Transwell chamber assay. Representative pictures shows the effects of miRNA-296-3p on migration and invasion. (a, b) Bar graph showing the migrated distance and number of migrated or invaded Huh-7 and HepG2 cells. (c) Western blot was used to evaluate migration- and invasion-related proteins (Cox-2, MMP-2, and MMP-9). Values were mean ± SE, ^*∗*^*P* < 0.05, ^*∗∗*^*P* < 0.01*vs.* control, *n* = 3 per group.

The effects of miRNA-296-3p on the migration and invasion capacities of HCC cells were investigated using wound healing assays, transwell assays, and western blot analysis. Transfection with miRNA-296-3p mimic reduced the wound closure rate of Huh-7 and HepG2 cells, as demonstrated in Figure 3(a). Furthermore, the transwell experiment revealed that the number of migrated/invaded cells was reduced compared to the control group, whereas miRNA-296-3p was overexpressed in these cells (Figure 3(b)). Finally, a similar conclusion was reached after detecting the expression of migrated-associated proteins (Cox-2, MMP-9, and MMP-2) using western blot analysis (Figure 3(c)). All of these findings revealed that miRNA-296-3p played a critical role in inhibiting HCC cell motility and invasion.

### 3.4. MSL2 Is a Target Gene of miRNA-296-3p

**Figure 4:**
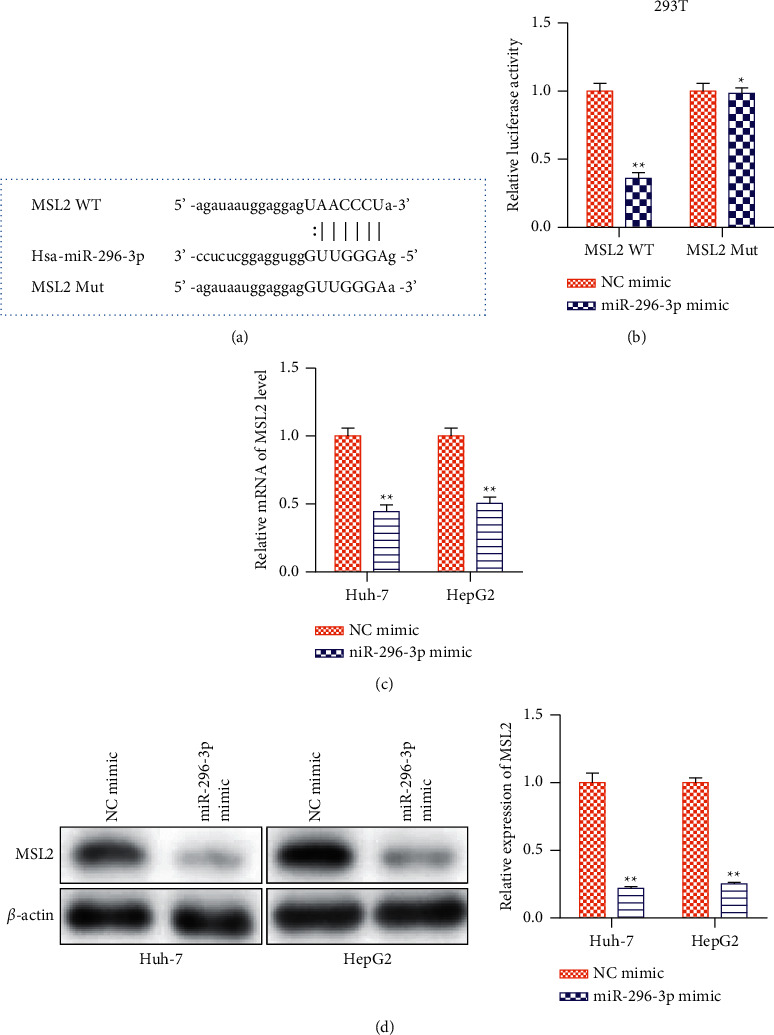
MSL2 is a target gene of miRNA-296-3p. (a) Bioinformatic analysis predicted complementary sequences between miRNA-296-3p and MSL2. The schematic diagram shows the putative binding sites of miRNA-296-3p on the 3′′-UTR region of MSL2. (b) Luciferase reporter assay suggested that miRNA-296-3p mimic reduced the luciferase activity of MSL2 WT in HEK-293T cells. WT: wild type; Mut: mutation. ^*∗∗*^*P* < 0.01*vs.* NC mimics, *n* = 3 per group. (c) mRNA and (d) protein levels of MSL2 in Huh-7 and HepG2 cells transfected with NC mimic or miRNA-296-3p mimic. Values were mean ± SE, ^*∗*^*P* < 0.05, ^*∗∗*^*P* < 0.01*vs*. control, *n* = 3 per group.

MSL2 has been proposed as a target for the therapy of HBV-related HCC in previous research [22]. To explore whether miRNA-296-3p inhibited HCC progression by targeting MSL2, we predicted the potential binding sites between miRNA-296-3p and MSL2 with the help of the TargetScan database. The 3′-UTR region of MSL2 mRNA had a binding site for miRNA-296-3p, which was discovered. A dual-luciferase reporter experiment in HEK-293T cells was used to further investigate the relationship between miRNA-296-3p and MSL2. When cells were transfected with miRNA-296-3p mimic, the luciferase activity of MSL2 3′-UTR was considerably reduced, as seen in Figure 4(b). However, when the putative binding sites were mutated, the miRNA-296-3p mimic exhibited modest effects. These findings determined that MSL2 was a target of miRNA-296-3p. Furthermore, we discovered that overexpression of miRNA-296-3p significantly altered MSL2 mRNA and protein levels in Huh-7 and HepG2 cells (Figures 4(c) and 4(d)). In Huh-7 and HepG2 cells, the amount of MSL2 was found to be inversely linked with miRNA-296-3p.

### 3.5. miRNA-296-3p Mediates on the HCC Progression by Regulating MSL2

**Figure 5:**
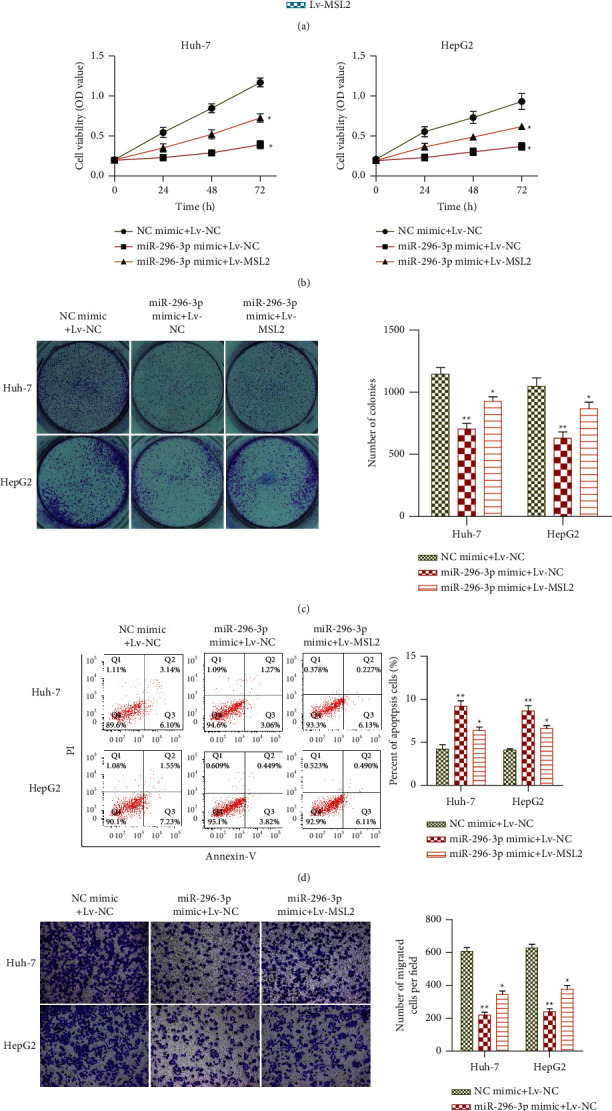
Effects of miRNA-296-3p on the HCC progression by regulating MSL2. Upregulation of MSL2 expression restored the inhibition effects of miRNA-296-3p overexpression on proliferation, migration, and invasion of HCC cells. (a) qRT-PCR analysis of the expression of MSL2 in Huh-7 and HepG2 cells transfected with Lv-NC or Lv-MSL2, ^*∗∗*^*P* < 0.01*vs.* Lv-NC. (b–e) Huh-7 and HepG2 cells were transfected with miRNA-296-3p mimic or/and Lv-MSL2. (b) CCK-8 was used to examine the cell viability. (c) Colony formation assay for the cell growth. (d) Flow cytometry analysis of the cell apoptosis rate. (e) Transwell chamber assay for the cell migration abilities. Values were mean ± SE, ^*∗*^*P* < 0.05 or ^*∗∗*^*P* < 0.01*vs.* NC mimic, ^#^*P* < 0.05*vs.* miRNA-296-3p mimic, *n* = 3 per group.

MSL2 was determined as a direct mediator of miRNA-296-3p in Huh-7 and HepG2 cells. However, the functional relationship between miRNA-296-3p and MSL2 remained unclear. To investigate whether miRNA-296-3p overexpression inhibited HCC progression through MSL2, we performed rescue experiments using miRNA-296-3p mimic and Lv-MSL2. Huh-7 and HepG2 cells were treated with NC mimic + Lv-NC, miRNA-296-3p mimic + Lv-NC, and miRNA-296-3p mimic + Lv-MSL2. In Huh-7 and HepG2 cells, the qRT-PCR experiment revealed that Lv-MSL2 dramatically increased MSL2 expression (Figure 5(a)). Upregulation of miRNA-296-3p dramatically reduced the viability of Huh-7 and HepG2 cells in the CCK-8 experiment. However, overexpression of MSL2 rescued the inhibition effect of miRNA-296-3p overexpression on cell proliferation (Figure 5(b)). Similarly, a colony formation experiment revealed that the number of colonies was reduced when these cells were transfected with miRNA-296-3p mimic. In contrast, cotransfection with miRNA-296-3p mimic and Lv-MSL2 restored this inhibition (Figure 5(c)). Furthermore, a flow cytometry analysis revealed that cells cotransfected with miRNA-296-3p mimic and Lv-MSL2 had a lower apoptosis rate than cells transfected with miRNA-296-3p mimic alone (Figure 5(d)). In transwell chamber assay, we observed similar rescue effects that the inhibitory effects of migration caused by miRNA-296-3p overexpression were reversed by MSL2 upregulation (Figure 5(e)). All of these findings showed that MSL2, a miRNA-296-3p target gene, reduced the effect of miRNA-296-3p overexpression on HCC cell proliferation, death, and migration.

## 4. Discussion

Currently, it remains a significant challenge to search for novel therapeutic strategies in HCC. Several improperly expressed miRNAs in HCC tumors and adjacent nontumorous tissues have been identified by miRNA profiling of HCC tumor tissues and adjacent nontumorous tissues [25–28]. Thus, exploring the relevant mechanisms between miRNA and HCC has become a novel strategy to find HCC therapy. miRNA-296-3p was found to be underexpressed in HCC cell lines in this investigation. miRNA-296-3p overexpression inhibited cell proliferation, migration, and invasion in a practical sense. All these data demonstrate that miRNA-296-3p possesses antitumor effects in HCC. Previous studies have shown that miR-296-5p reduces tumor growth and spread in HCC [19].

Increasing evidence has confirmed that miRNA-296-3p exerts beneficial effects on regulating the biological behavior of human cancer cells. miRNA-296-3p expression is reduced in malignant choroidal melanoma [29]. miRNA-296-3p inhibits tumor cell proliferation, invasion, and metastasis in lung adenocarcinoma (LADC) [18]. Moreover, miRNA-296-3p participates in suppressing clear cell renal cell carcinoma (ccRCC) metastasis [30]. Concerning the progression of HCC, the functions and molecular mechanisms of miRNA-296-3p are still poorly understood. miRNA-296-3p overexpression in HCC cell lines inhibited cell proliferation and migration/invasion but enhanced cell death, according to the findings. Furthermore, we discovered that miRNA-296-3p overexpression reduced MSL2 mRNA and protein levels in HCC cell lines [21, 22].

Male-specific lethal 2 (MSL2) exerts crucial roles in several molecular processes, such as targeting histone H2B and the tumor suppressor p53 [31]. MSL2 can enhance the growth of HepG2 cells *in vitro* and *in vivo* [23]. We used bioinformatics and a luciferase assay further to understand the mechanism of MSL2 on HCC cells. MSL2 was discovered to be a direct target of miRNA-296-3p, according to the findings. The level of MSL2 was shown to be adversely linked with miRNA-296-3p in our research. Overexpression of MSL2 decreased the effects of miRNA-296-3p overexpression on HCC cell proliferation, apoptosis, and migration, according to rescue experiments. It is well understood that p53's cytoplasmic location is essential for p53-mediated apoptosis and autophagy [31]. As a result, MSL2 functions as a critical ubiquitin ligase, mediating p53 ubiquitination and promoting p53 cytoplasmic localization. However, more research was needed to see if MSL acted as a regulator of p53-mediated apoptosis in HCC.

Taken together, we found that miRNA-296-3p inhibited the proliferation, migration, and invasion in HCC by targeting MSL2. Thus, MSL2 was validated as a novel target of miRNA-296-3p. Furthermore, our findings revealed the therapeutic goals of the novel HCC. They revealed that its clinical efficacy was worthy of further study.

## Data Availability

All data generated or analyzed during this study are included in this published article.

## Supplementary Materials

Supplementary Materials Original western blot: uncropped original picture of western blot.
