# Supplementary material for: In Hepatocellular Carcinoma, miRNA-296-3p Targets MSL2 and Suppresses Cell Proliferation and Invasion
Source: J Oncol. 2021 Dec 2;2021:7430468. doi: 10.1155/2021/7430468 (PMC8660223; doi:10.1155/2021/7430468)
Supplement: Supplementary Materials — Original western blot: uncropped original picture of western blot. [file 7430468.f1.pdf]

HUH-7

Cyclin D1(23)

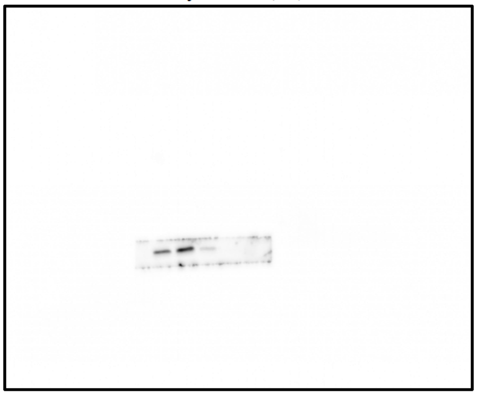

CDK2(23)

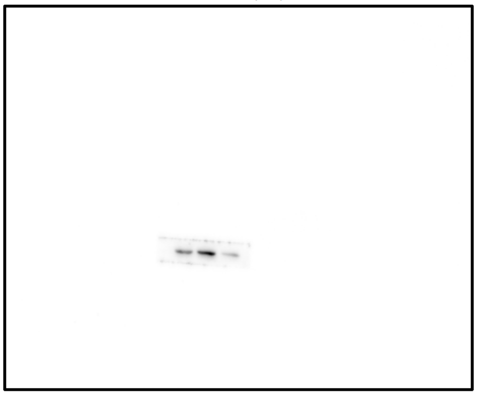

p21(12)

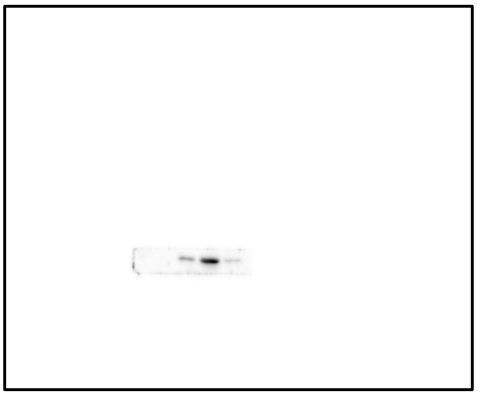

β-actin(12)

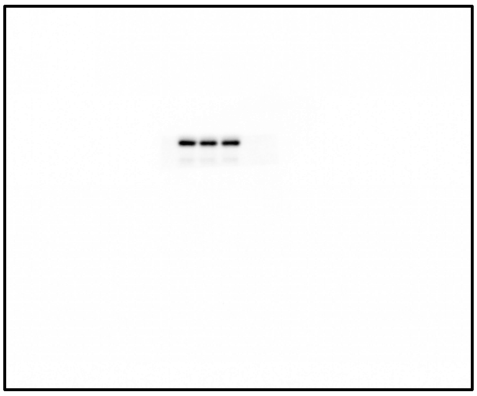

HepG2

Cyclin D1(23)

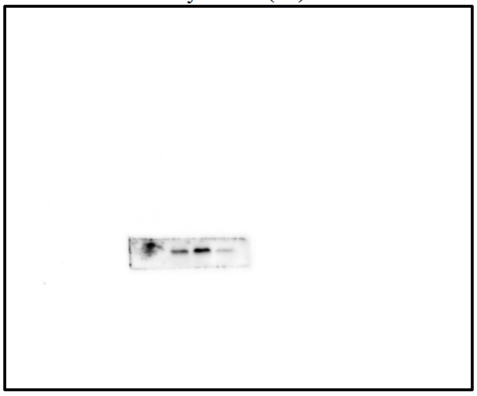

CDK2(23)

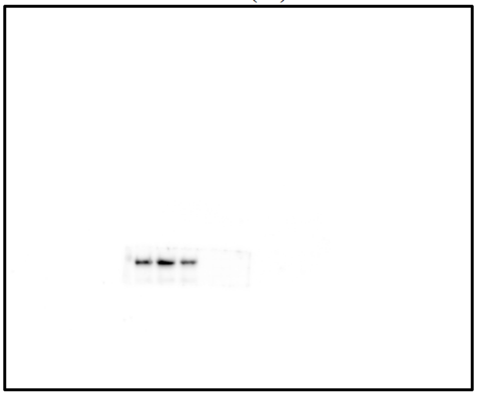

p21(23)

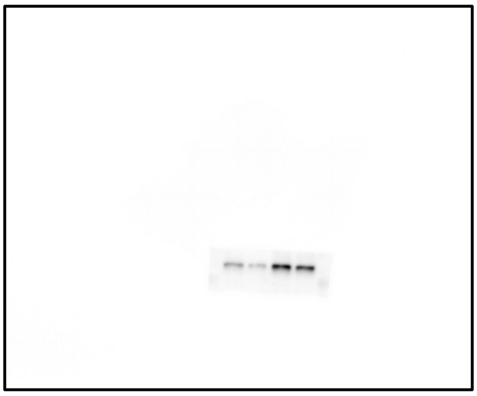

β-actin(12)

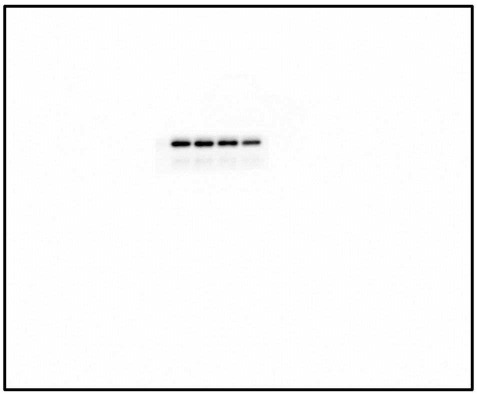

Uncropped original picture of Western blot inFigure 2C.

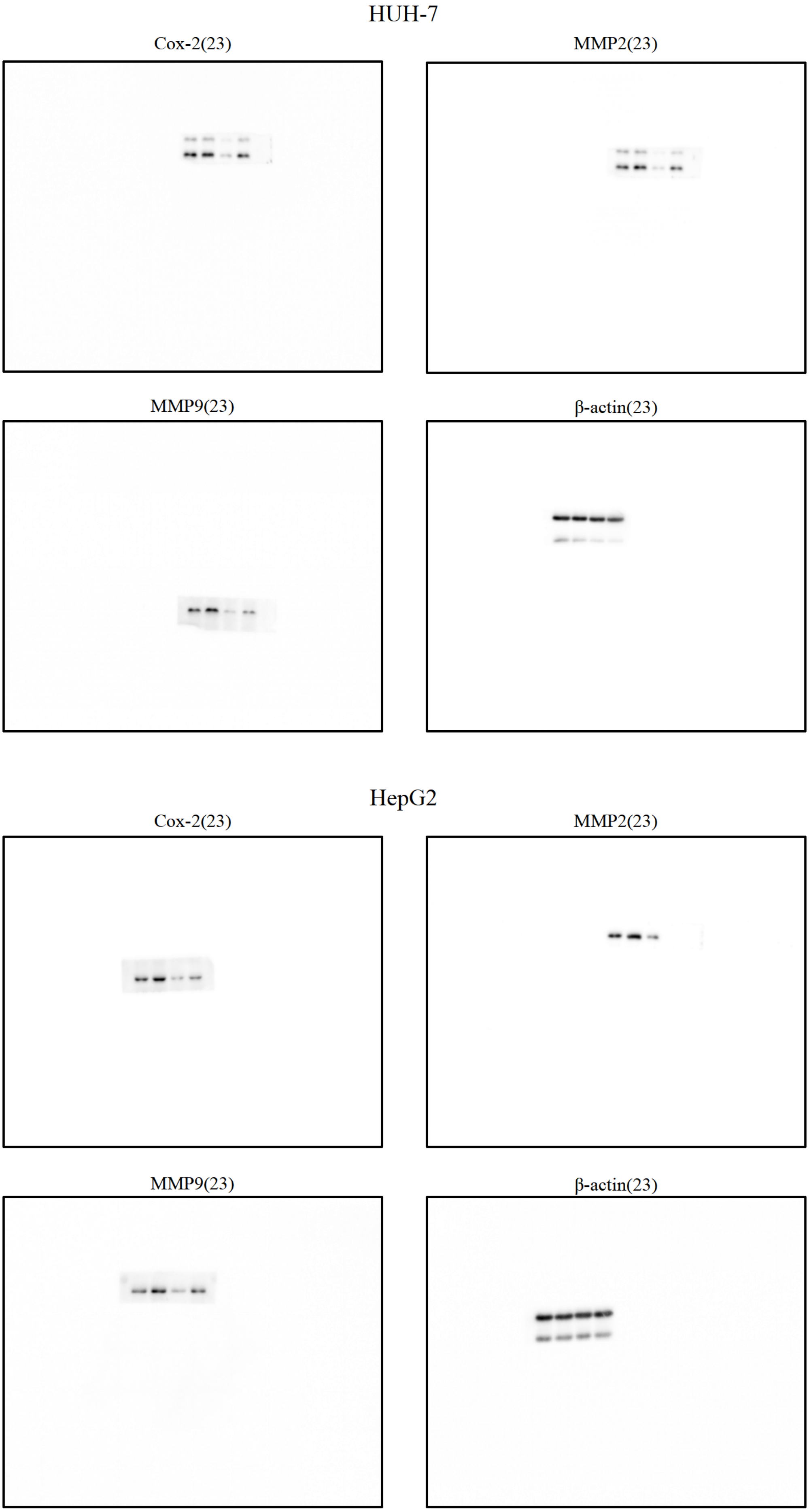

Uncropped original picture of Western blot inFigure 3C

HUH-7

MSL2(56)

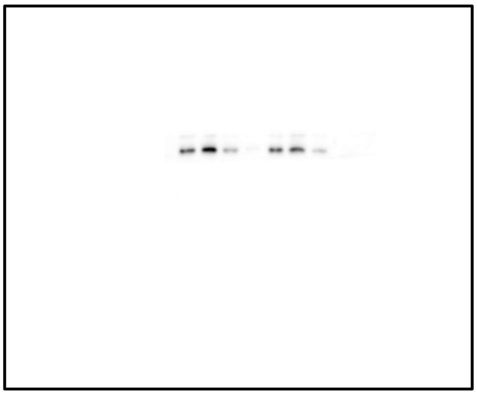

β-actin(23)

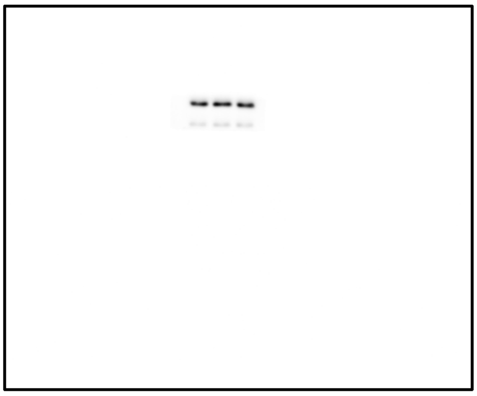

HepG2

MSL2(23)

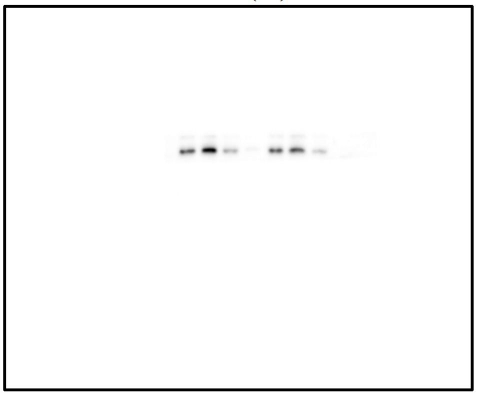

β-actin(12)

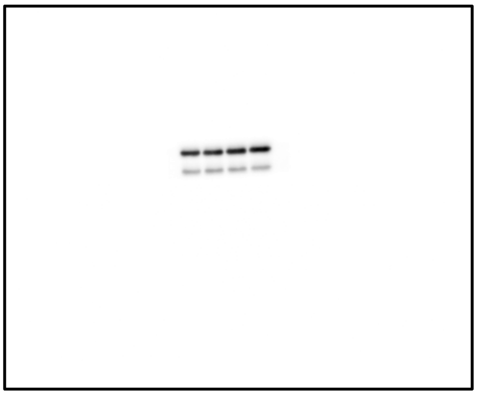

Uncropped original picture of Western blot inFigure 4D
